# Early Lung Function Testing in Infants with Aortic Arch Anomalies Identifies Patients at Risk for Airway Obstruction

**DOI:** 10.1371/journal.pone.0024903

**Published:** 2011-09-23

**Authors:** Charles Christoph Roehr, Silke Wilitzki, Bernd Opgen-Rhein, Karim Kalache, Hans Proquitté, Christoph Bührer, Gerd Schmalisch

**Affiliations:** 1 Department of Neonatology, Charité Universitätsmedizin Berlin, Berlin, Germany; 2 Department of Paediatric Cardiology, Charité Universitätsmedizin Berlin, Berlin, Germany; 3 Department of Obstetrics and Prenatal Medicine, Charité Universitätsmedizin Berlin, Berlin, Germany; Erasmus University Rotterdam, Netherlands

## Abstract

**Background:**

Aortic arch anomalies (AAA) are rare cardio-vascular anomalies. Right-sided and double-sided aortic arch anomalies (RAAA, DAAA) are distinguished, both may cause airway obstructions. We studied the degree of airway obstruction in infants with AAA by neonatal lung function testing (LFT).

**Patients and Methods:**

17 patients (10 RAAA and 7 DAAA) with prenatal diagnosis of AAA were investigated. The median (range) post conception age at LFT was 40.3 (36.6–44.1) weeks, median body weight 3400 (2320–4665) g. Measurements included tidal breathing flow-volume loops (TBFVL), airway resistance (R_aw_) by bodyplethysmography and the maximal expiratory flow at functional residual capacity (V′_max_FRC) by rapid thoracic-abdominal compression (RTC) technique. V′_max_FRC was also expressed in Z-scores, based on published gender-, age and height-specific reference values.

**Results:**

Abnormal lung function tests were seen in both RAAA and DAAA infants. Compared to RAAA infants, infants with DAAA had significantly more expiratory flow limitations in the TBFVL, (86% vs. 30%, p<0.05) and a significantly increased R_aw_ (p = 0.015). Despite a significant correlation between R_aw_ and the Z-score of V′_max_FRC (r = 0.740, p<0.001), there were no statistically significant differences in V′_max_FRC and it's Z-scores between RAAA and DAAA infants. 4 (24%) infants (2 RAAA, 2 DAAA) were near or below the 10^th^ percentile of V′_max_FRC, indicating a high risk for airway obstruction.

**Conclusion:**

Both, infants with RAAA and DAAA, are at risk for airway obstruction and early LFT helps to identify and to monitor these infants. This may support the decision for therapeutic interventions before clinical symptoms arise.

## Introduction

Aortic arc anomalies (AAA) are rare congenital cardiac anomalies; they represent approx. 1–3% of all cardiovascular anomalies [1]. In right-sided aortic arches (RAAA) the aorta originates from the left ventricle and takes a rightward turn before its descent through the mediastinum, whereas double-sided aortic arch anomalies (DAAA) are combinations of a right-sided and a left sided aortic arch [2]. This duplication often forms a vascular ring around the trachea and/or oesophagus, which tends to compress these [3], [4], [5]. Likewise, a RAAA, together with the ligamentum arteriosum (the remnant of the ductus arteriosus Botalli) may also cause compression of the mediastinal structures by forming a so called aortic sling. Therefore, both anomalies may cause stridor, cough, dyspnoea, dysphagia and recurrent lower airway infections [6], [7], [8], [9]. Based on surgical series, DAAA are the most common causes of vascular rings, followed by a right aortic arch with an aberrant left subclavian and left ductal ligament [10]. According to Bonnard et al. the majority (94%) of symptomatic AAA were successfully treated with surgical repair [8]. Hence, there is commonly a low threshold for early corrective surgery in these patients.

AAA can be identified by fetal ultrasound as early as 12 weeks gestational age (GA) [11]. The postnatal diagnosis of AAA can be made by echocardiography, barium swallow studies, magnetic resonance imaging (MRI), or computer tomography (CT) [12], [13]. While echocardiography allows sufficient imaging of the great vessels, airway obstruction is either confirmed by indirect imaging studies like barium swallow [14], or by CT or MRI, which are associated with either high doses of radiation and/or high costs [6]. Therefore, lung function tests (LFT) have been suggested as non-invasive, non-radiating diagnostic tests to diagnose and monitor the progression of airway obstruction in patients with AAA [15]. However, possibly due to lack of systematic investigations, only little data exists on LFT in children with AAA [15], [16].

We hypothesized that lung function testing in the neonatal period allows the identification and monitoring of patients with AAA. We aimed to investigate newborn infants with AAA and to describe the typical LFT findings.

## Methods

### Ethics statement

Neonatal lung function testing (LFT) was approved by the Charité University Hospital's ethics committee (ID 54/92). Written parental consent was obtained before each individual LFT.

### Subjects

This was a retrospective study from a large tertiary referral centre for congenital abnormalities (Charité University Medical Centre Berlin). All surviving neonates with a prenatal diagnosis of AAA, born between January 2005 and March 2011, were included in the study. Postnatal management included admission to the neonatal intensive care unit for observation of cardiac or respiratory symptoms and further diagnostic echocardiography.

### Protocol

Whenever possible, tests were performed around discharge from the unit. A single operator performed the LFTs in our lung function laboratory. Patients were not investigated if they had symptoms of an active respiratory tract infection (RTI), or had suffered a RTI within 2 weeks prior to the scheduled LFT. Before LFT, body weight was measured with a standard digital scale to the nearest 10 grams (Seca®, Hamburg, Germany), body length from crown to heel was measured by an inelastic tape to the nearest centimetre.

Infants were studied after having received chloral hydrate (50 mg kg^−1^) given orally 30–60 min before LFT. Sleeping infants were measured in supine position with the neck in a neutral position, supported by a neck roll. Three independent lung function tests were used to asses upper airway obstructions: the shape of the flow-volume measured during tidal breathing (TBFVL), the measurement of airway resistance using baby bodyplethysmography and the measurement of forced expiratory flow at functional residual capacity (V′_max_FRC).

**Figure 1:**
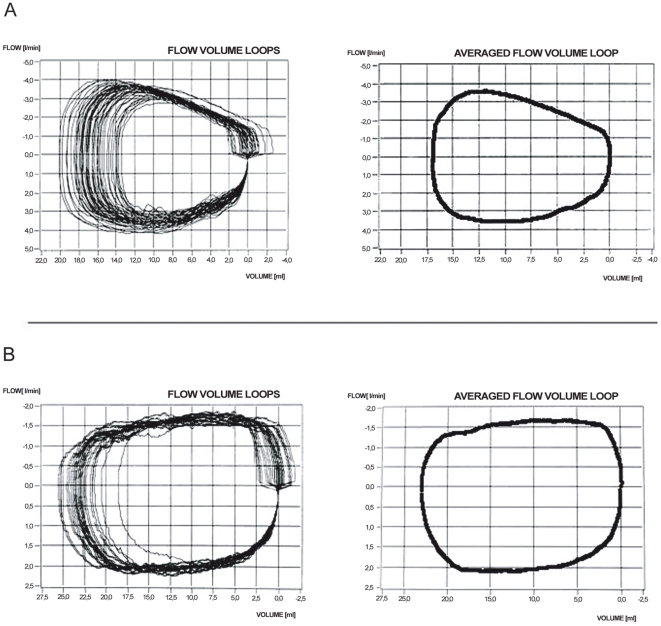
Consecutively measured tidal breathing flow-volume loops of newborns. Loops of a healthy newborn (1A) and loops of an infant with DAAA (1B), which illustrates the characteristic inspiratory and expiratory flow limitations. The left side of the figures show a series of consecutive breathing cycles and the right side the calculated averaged loop (by definition: inspiration shown on lower part of the graph, expiration on the top part of the graph).

TBFVLs were measured using custom made equipment based on the flow-through technique [17]. Briefly, the facemask is continuously rinsed thoroughly by a constant background flow, which eliminates apparatus dead space thus allowing long-term measurements. Depending on the variability of the breathing pattern 20–60 consecutive breaths were measured and an averaged breathing loop was calculated as described previously [18]. As shown in Fig. 1 upper-airway obstructions cause a horizontal flattening of the inspiratory and/or expiratory limb so that the TBFVL became a rectangular shape, as described by Leonhardt et al. [19].

The airway resistance (R_aw_) was measured by baby-bodyplethysmography (Jaeger Master Screen/Babybody, VIASYS, Würzburg, Germany) [20]. Upper airway obstructions cause an increase in R_aw_, however, the measured R_aw_ is highly method- and growth-dependent and there are no accepted reference ranges [21].

With the same equipment V′_max_FRC were measured using the rapid thoracic-abdominal compression (RTC) technique, according to international guidelines [22]. V′_max_FRC was also expressed in standard deviation scores (Z-scores) based on sex-, age and hight-specific reference values of healthy infants published by Hoo et al. [23].

During the pulmonary function tests, heart rate and oxygen saturation were monitored continuously by pulse oximetry (model N-200; Nellcor, Hayward, California).

### Statistical methods

Patient's characteristics and the results of LFT were described by incidences or median and range. For the comparison between infants with RAAA and DAAA the exact Fischer test or Mann-Whitney rank test was used as appropriate. Regression analysis was used to investigate the relationship between the Z-score of V′_max_FRC and R_aw_. Statistical analysis was performed using Statgraphics Centurion® software (Version 15.0, Statpoint Inc., Herndon, Virginia, USA). A level of statistical significance of p<0.05 was accepted.

## Results

### Patients

Seventeen patients with AAA (10 RAAA, 7 DAAA) were analysed. The median (range) post conceptional age at LFT was 40.3 (36.6–44.1) weeks and the median body weight was 3400 (2320–4665) grams. There was no statistically significant difference between the infants with RAAA or DAAA at birth or at the time of LFT, as shown in Table 1.

**Table 1:** Comparison of patient characteristics of infants with right-sided (RAAA) and double-sided aortic arch anomaly (DAAA) (presented as median (range) or n (%)).

	RAAA n = 10	DAAA n = 7	p-value
*At birth*			
Gestational age (weeks)	38.5 (36–41)	40 (36–41)	0.520
Birth weight (g)	3295 (2800–3665)	3420 (2330–4150)	0.435
Male	5 (50%)	2 (29%)	0.622
*At time of measurement*			
Age (days)	6.5 (3–22)	9 ( 5–34)	0.202
Post conceptional age (weeks)	39.4 (36.6–44.1)	40.9 (37.3–44.0)	0.305
Body weight (g)	3250 (2605–3780)	3550 (2320–4665)	0.353
Body length (cm)	50 (46–55)	53 (44–56)	0.224

### Lung function measurements

LFT were performed around the 40^th^ post conceptional week (term). The results are shown in table 2: The TBFVL indicated significantly more expiratory flow limitations in infants with DAAA compared to infants with RAAA. The combination of inspiratory and expiratory flow limitation was only seen in four infants with DAAA. Figure 1 illustrates a characteristic example of a typical TBFVL, as seen in DAAA patients.

**Table 2:** Comparison of the results of lung function testing in infants with right-sided and double-sided aortic arch anomaly (presented as median (range) or n (%), statistically significant p-values are printed in bold).

	RAAA n = 10	DAAA n = 7	p-value
*Shape of the TBFVL*			
Inspiratory flow limitation	2 (20%)	4 (57%)	0.162
Expiratory flow limitation	3 (30%)	6 (86%)	**0.049**
Combined inspiratory and expiratory flow limitation	0 (0%)	4 (57%)	**0.015**
*Measurements of airway resistance*			
R_aw_ (kPa/L/s)	1.58 (0.35–5.9)	3.09 (1.05–12.93)	0.097
R_aw_ (kPa/L/s) >2.81 kPa/L/s	2 (20%)	6 (86%)	**0.015**
*Measurement of V′_max_FRC*			
V′_max_FRC (mL/s)	95.5 (19–182)	75 (53–168)	0.435
Z-Score V′_max_FRC	−0.16 (−1.87–1.07)	−0.93 (−1,58–0.77)	0.283

**Figure 2:**
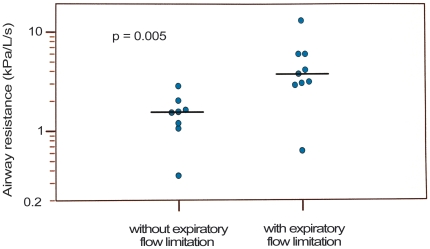
Expiratory flow limitation of the TBFVL and its impact on airway resistance (R_aw_ is presented on a logarithmical scale and the horizontal bar represents the median).

An expiratory flow limitation was associated with a significantly increased R_aw_ (Fig. 2). However, there were differences in R_aw_ between infants with RAAA and DAAA. As shown in table 2, 6/7 (86%) infants with DAAA had an increased R_aw_ (p = 0.015) above the group median of all infants (2.81 cmH_2_O/L/s).

**Figure 3:**
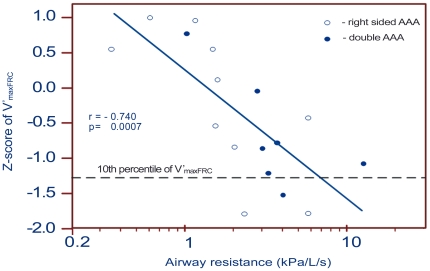
Correlation between Z-score of V′_max_FRC and R_aw_ of infants with AAA.

Despite a significant correlation between R_aw_ and the Z-score of V′_max_FRC (r = 0.740, p<0.001) there were no statistically significant differences in V′_max_FRC and the Z-scores of V′_max_FRC between RAAA and DAAA infants. As shown in Fig. 3, four (24%) infants (2 RAAA, 2 DAAA) were near or below the 10^th^ percentile of V′_max_FRC, which is indicative for a high risk of airway obstructions.

## Discussion

The study has shown that most infants with AAA have normal LFT at term. However, infants with DAAA as well as RAAA are at increased risk of airway obstruction, and these infants can clearly be identified by early LFT.

The risk of airway obstruction is higher in infants with DAAA compared to infants with RAAA. Combined in- and expiratory flow limitation (illustrated by the typical rectangular TBFVL, Fig. 1) was only seen in infants with DAAA. Most DAAA infants showed increased R_aw_ and their median R_aw_ was almost twice that of infants with RAAA, as shown in Table 2.

There is a lack of LFT studies in infants with AAA and this is to date the largest aggregation of early, pre-surgical lung function testing of such infants. Previously, Thomson et al. compared pre- and postoperative LFT in six children with vascular rings, also using a Jaeger Baby-Plethysmograph® [15]: All patients had pathologic LFT and evidence of tracheal compression; four infants had decreased values for FRC [15]. Also, and similar to our study, Amirav et al. found significantly increased R_aw_ and decreased values for V′_max_FRC [16].

To date, the gold standard for confirming the diagnosis of AAA is by contrast CT or MRI [12]. Comparative studies of LFT and imaging studies have not yet been performed. Performing LFT in AAA offers the advantage of using a non-invasive method, which spares the patient a high load of radiation (CT) or intensive apparatus and cost generating investigations (MRI/CT). We believe that early LFT can serve as a non-invasive tool for diagnosing the extent of airway compression by the shape of the TBFVL, the elevation in R_aw_ and V′_max_FRC reduction.

The decision for corrective surgery is traditionally based on the patient's clinical presentation. However, one patient from our analysis with a preoperative diagnosis of RAAA was intra-operatively found to have DAAA. Interestingly, this patient had a longstanding history of >3 clinical symptoms related to AAA, including recurrent respiratory infections. The TBFVL at term showed the typical configuration of a DAAA infant. His R_aw_ at term (3.71 cmH_2_O/L/s) was higher than the median found in our RAAA group (1.58 cmH_2_O/L/s) and even higher than that for the DAAA group (3.09 cmH_2_O/L/s). Also, his V′_max_FRC of 64 L/s was well below the median (95.5 L/s) found for RAAA infants.

Three neonatal lung function tests were used in this study. These tests assessed different aspects of airway obstruction. The TBFVL demonstrates flow limitations, which are predictive for airway obstruction [19]. However, TBFVL have the disadvantage that they not only reflect lung mechanics but also the patients' voluntary breathing patterns [24]. As for the other tests, R_aw_ primarily describes the conductivity of the upper airways, while V_max_FRC mostly describes small airway conductivity [22]. Our study has shown that the combination of these three methods can reliably identify the presence and the degree of airway obstruction in AAA patients.

Our study has several limitations. Firstly, RAAA and DAAA are rare malformations. The small number of patient data in our study limits its statistical power and increases the risk of a type II error. Only a limited number of measurements were available for comparing subgroups. Thus, the absence of significant differences in R_aw_ and V_max_FRC between both patient groups may be due to the low sample size and the high inter subject variability of lung function parameters. Furthermore, to date no age-dependent reference values exist for R_aw_, mainly because these values are highly dependent on the method of measurement and of the infants' growth [21], [25]. Although we believe to have found a clinically useful interpretation of R_aw_ by using the relationship between R_aw_ and the Z-score of V′_max_FRC, this has not been previously studied and requires further investigation.

In conclusion, and different to previous common belief, we found that both groups of AAA patients (RAAA and DAAA) are at risk for airway obstruction. Early LFT helps to identify and to monitor such infants. Early LFT may support the decision for a surgical intervention even before clinical symptoms arise.

## References

[1] Weinberg PM, Allen, Gutgesell Clark Driscoll (eds) (2001). Aortic arch anomalies.. Moss and Adam's heart disease in Infants, Children and Adolescents, 6th edition.

[2] Marmon LM, Bye MR, Haas JM, Balsara RK, Dunn JM (1984). Vascular rings and slings: long-term follow-up of pulmonary function.. Journal of Pediatric Surgery.

[3] Eichhorn JG, Fink C, Delorme S, Ulmer H (2004). Rings, slings and other vascular abnormalities: ultrafast computed tomography and magnetic resonance angiography in pediatric cardiology.. Zeitschrift für Kardiologie.

[4] Hernanz-Schulman M (2005). Vascular rings: a practical approach to imaging diagnosis.. Pediatric Radiology.

[5] McLaren CA, Elliott MJ, Roebuck DJ (2008). Vascular compression of the airway in children.. Pediatric Respiratory Reviews.

[6] Turner A, Gavel G, Coutts J (2005). Vascular rings–presentation, investigation and outcome.. Eur J Pediatr.

[7] Griffiths AL, Massie J, South M (2005). Double aortic arch presenting as severe bronchiolitis in a 2-week-old infant.. J Paediatr Child Health.

[8] Bonnard A, Auber F, Fourcade L, Marchac V, Emond S (2993). Vascular ring abnormalities: a retrospective study of 62 cases.. Journal of Pediatric Surgery.

[9] Lowe GM, Donaldson JS, Backer CL (1991). Vascular rings: 10-year review of imaging.. Radiographics.

[10] Dodge-Khatami A, Tulevski II, Hitchcock JF, de Mol B, Bennink GE (2002). Vascular rings and pulmonary arterial sling: from respiratory collapse to surgical cure, with emphasis on judicious imaging in the hi-tech era.. Cardiology in the young.

[11] Zidere V, Tsapakis EG, Huggon IC, Allan LD (2006). Right aortic arch in the fetus.. Ultrasound Obstet Gynecol.

[12] Ma GQ, Li ZZ, Li XF, Peng Y, DU ZD (2007). Congenital vascular rings: a rare cause of respiratory distress in infants.. Chinese Medical Journal.

[13] Humphrey C, Duncan K, Fletcher S (2006). Decade of experience with vascular rings at a single institution.. Pediatrics.

[14] Burch M, Balaii S, Deanfield JE, Sullivan ID (1993). Investigation of vascular compression of the trachea: the complementary roles of barium swallow and echocardiography.. Arch Dis Child.

[15] Thomson AH, Beardsmore CS, Firmin R, Leanage R, Simpson H (1990). Airway function in infants with vascular rings: preoperative and postoperative assessment.. Arch Dis Child.

[16] Amirav I, Rotschild M, Bar-Yishay E (2003). Pulmonary function tests leading to the diagnosis of vascular ring in an infant.. Pediatric Pulmonology.

[17] Schmalisch G, Foitzik B, Wauer RR, Stocks J (2001). Effect of apparatus dead space on breathing parameters in newborns: “flow-through” versus conventional techniques.. Eur Respir J.

[18] Schmalisch G, Schmidt M, Foitzik B (2001). Novel technique to average breathing loops for infant respiratory function testing.. Med Biol Eng Comput.

[19] Leonhardt S, Ahrens P, Kecman V (2010). Analysis of tidal breathing flow volume loops for automated lung-function diagnosis in infants.. IEEE Trans Biomed Eng.

[20] Bisgaard H, Nielsen KG (2005). Plethysmographic measurements of specific airway resistance in young children.. Chest.

[21] Stocks J, Godfrey S, Beardsmore C, Bar-Yishay E, Castile R (2001). Plethysmographic measurements of lung volume and airway resistance. ERS/ATS Task Force on Standards for Infant Respiratory Function Testing. European Respiratory Society/American Thoracic Society.. Eur Respir J.

[22] Sly PD, Tepper R, Henschen M, Gappa M, Stocks J (2000). Tidal forced expirations. ERS/ATS Task Force on Standards for Infant Respiratory Function Testing. European Respiratory Society/American Thoracic Society.. Eur Respir J.

[23] Hoo AF, Dezateux C, Hanrahan JP, Cole TJ, Tepper RS (2002). Sex-specific prediction equations for Vmax(FRC) in infancy: a multicenter collaborative study.. Am J Respir Crit Care Med.

[24] Schmalisch G, Wilitzki S, Wauer RR (2005). Differences in tidal breathing between infants with chronic lung diseases and healthy controls.. BMC Pediatrics.

[25] Stocks J, Coates A, Bush A (2007). Lung function in infants and young children with chronic lung disease of infancy: The next steps?. Pediatr Pulmonol.

